# Magnetic anchor technique assisted laparoscopic cholecystectomy in swine

**DOI:** 10.1038/s41598-023-32157-8

**Published:** 2023-03-24

**Authors:** Miaomiao Zhang, Jia Ma, Jingci Gai, Zhixuan Zhang, Haohua Wang, Yuhan Zhang, Yuxiang Ren, Yi Lyu, Xiaopeng Yan

**Affiliations:** 1 Department of Hepatobiliary Surgery, The First Affiliated Hospital of Xi’an Jiaotong University, 277 West Yanta Road, Xi’an, 710061 Shaanxi China; 2 National and Local Joint Engineering Research Center of Precision Surgery & Regenerative Medicine, The First Affiliated Hospital of Xi’an Jiaotong University, Xi’an, Shaanxi China; 3 Department of Surgical Oncology, Shaanxi Provincial People’s Hospital, Xi’an, Shaanxi China; 4 Qide College, Xi’an Jiaotong University, Xi’an, Shaanxi China; 5 Zonglian College, Xi’an Jiaotong University, Xi’an, Shaanxi China

**Keywords:** Gall bladder disease, Surgery, Biliary tract

## Abstract

Magnetic anchor device based on the principle of magnet heteropolar attraction can assist laparoscopic surgery and reduce abdominal wall trauma. This study explored the feasibility of use of our self-designed magnetic anchor device for reduced-port laparoscopic cholecystectomy (LC) through animal experiments. Twelve experimental pigs (15–20 kg) were randomly divided into study group (magnetic anchor technique assisted 2-port LC, n = 6) and control group (conventional 3-port LC, n = 6). Operative time, intraoperative blood loss, and postoperative complications were compared between the two groups. LC was successfully performed in all 12 pigs. There was no significant between-group difference with respect to operative time (study group: 35.83 ± 5.12 min; control group: 34.50 ± 5.13 min, P = 0.662) or intraoperative blood loss (< 50 mL per animal in both groups). In the experimental group, there was no malfunction of the magnetic anchoring device, the use process was smooth, and the tissue traction and surgical field exposure were satisfactory. There were no perioperative complications such as bile duct injury, bile leakage, or bleeding in both groups. We demonstrated the feasibility of use of the self-designed magnetic anchor device in reduced-port LC. The device has important clinical application value.

## Introduction

Minimally-invasive surgery is an important trend in future surgical development. The emergence of laparoscopic cholecystectomy (LC) was an important milestone in the development of minimally-invasive abdominal surgery^1^. Compared with traditional open cholecystectomy, LC offers the advantages of lesser trauma and quicker recovery. Indeed, LC is currently the standard surgical method for benign gallbladder diseases such as gallstones and gallbladder polyps^2^. Over the years, LC has evolved from the original 4-port to the currently popular 3-port approach^3^. Reduced-port surgery including 2-port and single-port are poised to become the future direction of development of LC^4,5^. However, reduced number of ports in the abdominal wall increases the difficulty of operation and reduces the flexibility^6^, which is a bottleneck restricting the development of reduced-port LC.

Magnetosurgery/magnetic surgery (MS), represented by magnetic compression technique (MCT) and magnetic anchor technique (MAT), offers a distinct leverage as a new surgical technology in clinical practice. The research on magnetosurgery was pioneered by Japanese scholars Obora et al. in 1978 in their study on magnetic vascular anastomosis^7^. The specially-designed magnetic anastomosis device can be used for vascular anastomosis^8–11^, digestive tract anastomosis^12–16^, therapeutic fistula^17^, and tracheoesophageal fistula animal model preparation^18^. In 2007, Park et al. proposed the concept of use of magnetic force to assist laparoscopic surgery in order to reduce the number of abdominal wall ports^19^. Subsequently, various magnetic anchor devices have been used in laparoscopic and thoracoscopic surgery^20–25^. These devices can help achieve better tissue traction and effectively reduce the inconvenience caused by the chopstick effect under the condition of reduced ports. In this study, we designed the magnetic anchor device by ourselves and validated the feasibility of use of the device for reduced-port LC in experimental pigs.

## Materials and methods

### Ethics statement

The ethics committee of the Xi’an Jiaotong University approved this study (permit number: 2022-1457). All animal experiments complied with the ARRIVE guidelines and were carried out in accordance with the National Institutes of Health Guide for the Care and Use of Laboratory Animals (eighth edition, 2011). The animal protocol was designed to minimize discomfort to the animals.

### Study design

We obtained 12 swine (6 males and 6 females; weight, 15–20 kg) from the Laboratory Animal Center of the Xi’an Jiaotong University (Xi’an, China). The pigs were randomly assigned to the study group (n = 6) or the control group (n = 6). In the study group, 2-port LC was performed using magnetic anchor device, while the conventional 3-port LC was performed in the control group. To eliminate research bias caused by human factors, the LCs of all 12 animals were performed by the same surgeon and first assistant. The surgeon was the attending physician Xiaopeng Yan, who has independently completed more than 300 cases of LC in clinic. And the first surgical assistant (magnet bearer) was Miaomiao Zhang.

### Magnetic anchor device

**Figure 1:**
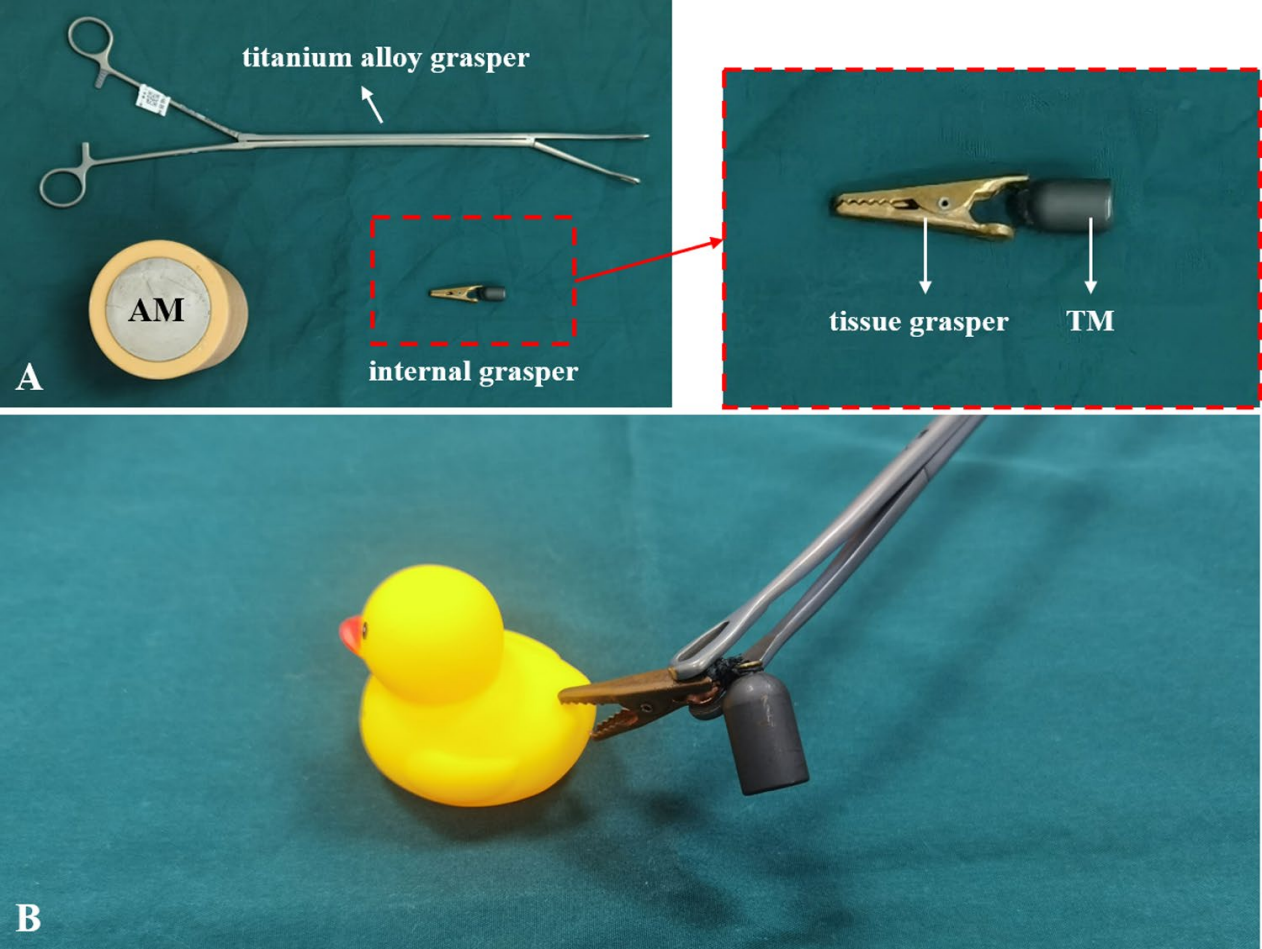
The magnetic anchor device. **(A)** Titanium alloy grasper, anchor magnet and internal grasper. **(B)** Titanium alloy grasper assists the internal grasper to clamp objects.

**Figure 2:**
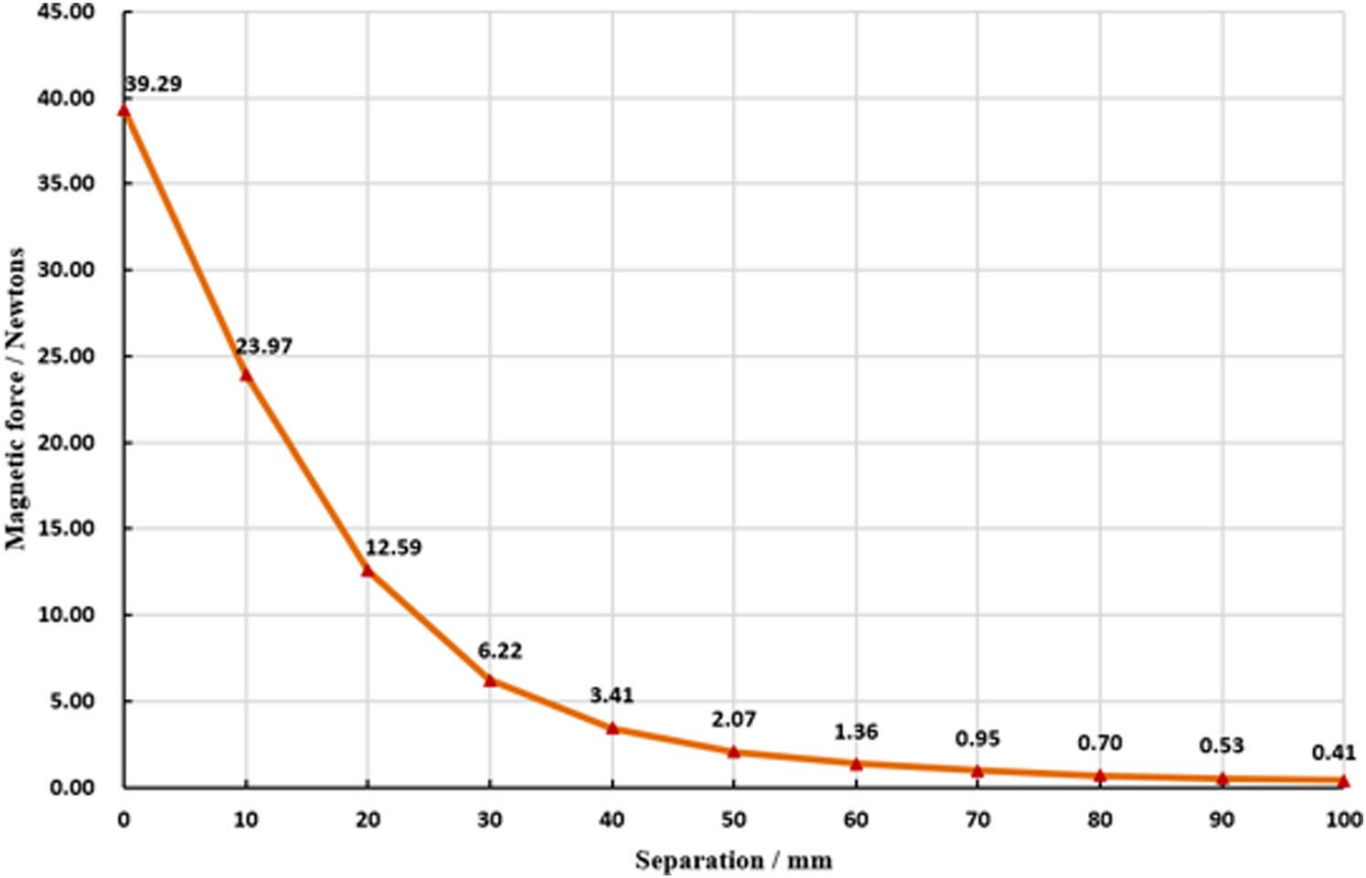
Magnetic force curve of the anchor magnet and the target magnet.

The magnetic anchor device includes two parts in vivo and in vitro. The external part is called the anchor magnet (AM), which is a cylindrical magnet with a diameter of 60 mm and a height of 160 mm. It is made of N45 sintered NdFeB, and the surface of the magnet is nickel-plated. The AM is covered with a plastic shell. The internal part is the internal grasper, the head end of the internal grasper is a tissue grasper made of copper material, and the tail end is the target magnet (TM). The TM is a cylinder with a diameter of 10 mm and a height of 15 mm, which is made of N50 sintered NdFeB, and is covered with a U-shaped metal shell. The entire internal grasper is approximately 50 mm long and can smoothly pass through the 12-mm Trocar. The AM weighed 2242 g, and had a magnetic density of 0.75 T. The internal grasper weighted 11.50 g, and the TM had a magnetic density of 0.53 T on the working surface. The third part of the magnetic anchor device is a titanium alloy grasper. The primary function of titanium alloy grasper is to clamp the internal grasper in the abdominal cavity and adjust the position of the internal grasper on the gallbladder. Titanium alloy grasper is made of titanium alloy and can be used to avoid attracting the target magnets. This is an important part of the magnetic anchor device, which is not available in conventional grasper. The magnetic anchor device is shown in Fig. 1. The maximum magnetic force of anchor magnet and target magnet at zero distance is 39.29 Newtons, and the magnetic force gradually decreases with the increase in the distance between the AM and TM. The magnetic force curve is shown in Fig. 2.

### Surgical procedures

After being fasted for 12 h, the pigs were weighed and then anesthetized using an intravenous injection of 3% pentobarbital sodium solution (1 mL/kg). After successful anesthesia, the experimental pigs were fixed in supine position, and subjected to tracheal intubation and ventilator-assisted ventilation. A 10-mm trocar was established under the umbilicus for entry of the laparoscopic light source and camera system. A 12-mm trocar under the xiphoid was established and the hook cautery was inserted. Adjust the body position to raise the head and the right side of the body. Due to the effect of gravity, the internal organs shift to the left and lower to expose the gallbladder.

**Figure 3:**
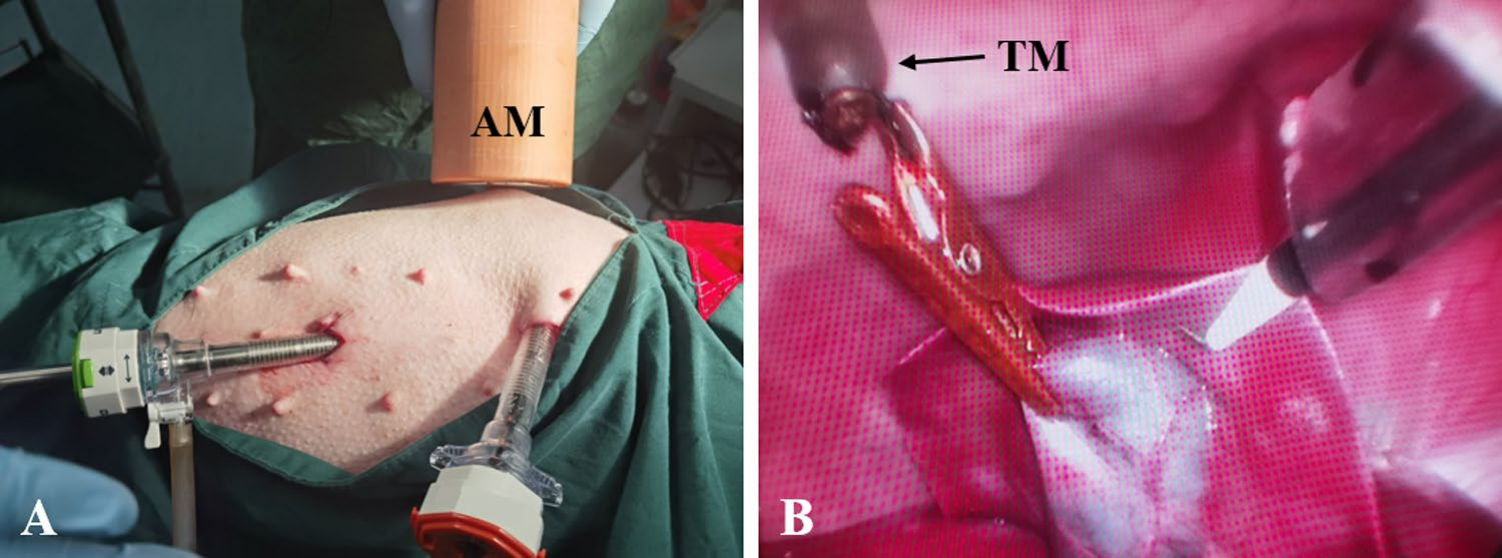
Intraoperative photographs showing the use of the magnetic anchor device. **(A)** Abdominal wall port and anchor magnet. **(B)** State of the internal grasper pulling the gallbladder.

Study group: the internal grasper was inserted through the 12-mm trocar, and the tissue grasper was clamped to the gallbladder with the assistance of titanium alloy grasper. The anchor magnet was placed outside the right upper abdominal wall of the pig (Fig. 3A). At this time, the target magnet at the end of the internal grasper was attracted by the anchor magnet (Fig. 3B). Attracted by the anchor magnet, the bottom of the gallbladder is pulled up to expose the triangular area of the gallbladder. In this process, the pulling direction and pulling force of the internal grasper can be changed by adjusting the position of the anchor magnet. The gallbladder triangle was dissected, and the cystic artery and cystic duct were clipped and cut. The titanium alloy grasper was held at the tail end of the tissue grasper to make the head end of the tissue grasper open. According to the requirements of the operation, the tissue grasper was clamped at the ampulla of the gallbladder, and the anchor magnet was moved to fully expose the gallbladder bed and maintain appropriate tissue tension. During the operation, a good operative field and tissue tension can be obtained by readjusting the clamping position of the tissue grasper on the gallbladder. The gallbladder was completely dissected from the gallbladder bed with a hook cautery. Anchor magnet was moved away, the internal grasper and the gallbladder through the 12-mm incision were removed with the assistance of the titanium alloy grasper.

Control group: a 5-mm trocar in the lower edge of the right costal arch was used to insert the spring grasping forceps. The difference from the study group was that in the control group, the spring grasping forceps was used instead of magnetic anchor devices to retract the gallbladder.

### Calculation of operation time

The operation time was defined as the time required for cholecystectomy, and was calculated from the beginning of the establishment of the abdominal wall port to the complete removal of the gallbladder and suture of the incisions. The operation time was recorded for each swine.

### Postoperative care

Each pig was housed individually after surgery. After recovery from anesthesia, there were no restrictions on food and water intake. During the first 3 days after surgery, intramuscular pethidine (1 mg/kg) was injected every 12 h. The mental state, feeding status, and activity of each pig were observed daily after operation, and the observation end point was 2 weeks after operation.

### Statistical analysis

Data were analyzed using the SPSS statistical software package (v19.0). The normality of distribution of continuous variables was assessed using the Shapiro–Wilk test. Normally distributed continuous variables are reported as mean ± standard deviation (SD) and between-group differences were assessed using the t test. Non-normally distributed continuous variables are reported as medians and interquartile range and between-group differences assessed using nonparametric tests. Categorical variables are reported as frequency and percentage, and were compared using Chi-squared or nonparametric tests, as appropriate. P values < 0.05 were considered indicative of statistical significance.

## Results

### Procedural parameters

LC was successfully performed in all pigs in both groups. There was no significant between-group difference with respect to operation time (study group: 35.83 ± 5.12 min; control group: 34.50 ± 5.13 min, P = 0.662). In the study group, the magnetic anchoring device was used smoothly, and there were no instances of device failure. There was no biliary tract injury, intestinal injury, or major intraoperative bleeding in any of the operations. The intraoperative blood loss did not exceed 50 mL in any of the pigs. The perioperative related indicators of the study group and the control group are shown in Table 1.

**Table 1:** Comparison between the study group and the control group.

	Study group	Control group	*P* value
Operation time (min)	35.83 ± 5.12	34.50 ± 5.13	0.662
Blood loss (mL)	28.33 ± 7.53	23.33 ± 12.11	0.411
Biliary tract injury (n)	0	0	–
Intestinal injury (n)	0	0	–
Degree of convenience^a^ (score)	8.17 ± 1.17	8.83 ± 0.75	0.267
Satisfaction of surgical field exposure^b^ (score)	6.83 ± 0.75	7.83 ± 1.17	0.109
Postoperative bilirubin level			–
Normal (n)	6	6	
Abnormal (n)	0	0	

^a^The degree of convenience was scored by the operator according to the operation feeling, and the score was limited to 0 to 10 scores. The higher the score, the better the convenience.

^b^Satisfaction of surgical field exposure was scored by the operator according to the operation feeling, and the score was limited to 0 to 10 scores. Higher scores indicate better surgical field exposure.

### Survival rate and postoperative complications

Postoperative survival rate was 100% in both groups. The postoperative mental state was good, and the food intake on the third postoperative day was comparable to the preoperative state. None of the pigs in this study showed incisional infection, incisional hernia, sclera icteric, yellow urine, or fever up to 14 days after operation.

## Discussion

A number of methods and devices are used to assist retraction in reduced-port laparoscopic surgery^26–28^; however, these methods offer less flexibility. Magnets have the unique ability to attract each other without contact. The design and functionality of the magnetic anchor device leverage this unique feature of magnets. When using the magnetic anchor device, the pulling direction and pulling force of the internal grasper can be changed by adjusting the position of the anchor magnet. In the experiment, we found that use of magnetic force to stretch the gallbladder offered greater flexibility in the stretching direction, which is not available with other methods.

The magnetic anchor device designed in this study has the following characteristics: (1) Both the anchor magnet and the target magnet are cylinders, which are made of NdFeB materials, in order to obtain the largest magnetic force with the smallest magnet volume. As shown in the magnetic force curve, the maximum magnetic force between the anchor magnet and the target magnet can reach 39.29 Newtons at zero distance, and the magnetic force at 5 cm distance is 2.07 Newtons. Such a magnetic force can meet the needs of most patients. (2) The outer surface of the target magnet is covered with a U-shaped metal shell. Its’ main purpose is to shield and guide the magnetic field lines of the non-working surface of the target magnet to the working surface in order to increase the magnetic field strength of the working surface. Moreover, it also reduces the magnetic field strength of the non-working surface, thereby reducing the interference of the magnetic force with the surgical instruments during the operation. (3) The magnetic anchor device is ingenious in design, simple in structure, and convenient to use.

In this study, LC was successfully completed in both groups. The operation time in the study group was slightly longer than that in the control group, but the between-group difference was not statistically significant. The results showed that the convenience and field exposure satisfaction scores of magnetic anchor technique assisted 2-port LC were lower than those of conventional 3-port LC, but there was no statistical difference (*P* > 0.05). During the actual operation, we have to admit that the pulling effect of the magnetic anchor device cannot surpass the pulling effect of the spring grasping forceps used in the conventional operation. Therefore, the use of magnetic anchor device for reduced-port LC also requires a certain amount of surgical dexterity, which has a certain learning curve. During the application process of magnetic anchor devices, it is necessary to adjust the position of the anchor magnet to obtain a good pulling effect. Moreover, the clamping position of the tissue grasper on the gallbladder should be adjusted in time according to the needs. Satisfactory results can only be achieved by combining these two aspects organically and flexibly. However, there was no significant difference in blood loss or injure between the two groups. As a new surgical method, magnetic anchor technique assisted 2-port LC also has a certain learning curve. We believe that after the surgeon has completed the learning curve, intraoperative flexibility will be further improved and intraoperative complications will be safely resolved.

A limitation of this study was that the selected experimental pigs have smaller body weights and thinner abdominal walls, which is quite different from the abdominal wall of humans. In further research, experimental groups with different body weights can be selected to explore the feasibility of use of the magnetic anchor device. Furthermore, the magnetic anchor device can be used not only for gallbladder traction, but also for other abdominal organs such as appendix, liver, and stomach. Therefore, the application potential of magnetic anchor device should be explored in other laparoscopic surgeries.

In conclusion, the results of the study demonstrate the feasibility of use of our self-designed magnetic anchor device in reduced-port LC. The device has potential application prospects for clinical use.

## Data Availability

The datasets used and analyzed during the current study available from the corresponding author on reasonable request.
